# Immunosuppressive SOX9‐AS1 Resists Triple‐Negative Breast Cancer Senescence Via Regulating Wnt Signalling Pathway

**DOI:** 10.1111/jcmm.70208

**Published:** 2024-11-17

**Authors:** Xuan Ye, Yi Cen, Quan Li, Yuan‐Ping Zhang, Qian Li, Jie Li

**Affiliations:** ^1^ Department of Breast and Thyroid Surgery, Guangzhou Women and Children's Medical Center, Guangzhou Medical University Guangdong Provincial Clinical Research Center for Child Health Guangzhou PR China; ^2^ Guangdong Provincial Key Laboratory of Molecular Target & Clinical Pharmacology, the NMPA and State Key Laboratory of Respiratory Disease Guangzhou Medical University Guangzhou PR China

**Keywords:** immune infiltration, senescence, SOX9‐AS1, triple‐negative breast cancer, Wnt signalling pathway

## Abstract

Long noncoding RNAs (lncRNAs) are involved in the regulation of triple‐negative breast cancer (TNBC) senescence, while pro‐carcinogenic lncRNAs resist senescence onset leading to the failure of therapy‐induced senescence (TIS) strategy, urgently identifying the key senescence‐related lncRNAs (SRlncRNAs). We mined seven SRlncRNAs (SOX9‐AS1, LINC01152, AC005152.3, RP11‐161 M6.2, RP5‐968 J1.1, RP11‐351 J23.1 and RP11‐666A20.3) by bioinformatics, of which SOX9‐AS1 was reported to be pro‐carcinogenic. In vitro experiments revealed the highest expression of SOX9‐AS1 in MDA‐MD‐231 cells. SOX9‐AS1 knockdown inhibited cell growth (proliferation, cycle and apoptosis) and malignant phenotypes (migration and invasion), while SOX9‐AS1 overexpression rescued these effects. Additionally, SOX9‐AS1 knockdown facilitated tamoxifen‐induced cellular senescence and the transcription of senescence‐associated secretory phenotype (SASP) factors (IL‐1α, IL‐1β, IL‐6 and IL‐8) mechanistically by resisting senescence‐induced Wnt signal (GSK‐3β/β‐catenin) activation. Immune infiltration analysis revealed that low SOX9‐AS1 expression was accompanied by a high infiltration of naïve B cells, CD8^+^ T cells and γδ T cells. In conclusion, SOX9‐AS1 resists TNBC senescence via regulating the Wnt signalling pathway and inhibits immune infiltration. Targeted inhibition of SOX9‐AS1 enhances SASP and thus mobilises immune infiltration to adjunct TIS strategy.

## 1 Introduction

Triple‐negative breast cancer (TNBC) is the most malignant type of breast cancer, accounting for approximately 20% of the total [1]. Typically characterised by a lack of oestrogen receptor (ER), progesterone receptor (PR) and human epidermal growth factor receptor 2 (HER2), TNBC is characterised by a higher degree of aggressiveness, tumour burden and poorer prognosis compared with other subtypes [2]. Radiotherapy and chemotherapy are still the mainstay for TNBC therapy, which inevitably lead to resistance [3]. Interestingly, when low drug concentration for chemotherapy or low radiation intensity for radiotherapy is used, it can induce cellular senescence to control tumour growth, based on which the anti‐tumour strategy of therapy‐induced senescence (TIS) has been derived [4].

TIS is a safety and security response for cancer cells through DNA damage‐induced cellular senescence primarily without executing apoptosis [5]. TIS reflects a more complex cell autonomy and also generates non‐cell autonomous secretory phenotypes like senescence‐associated secretion phenotype (SASP) that collectively influence tumour progression [6, 7, 8]. However, increasing evidence suggests that partial oncogenes are involved in the regulation of cellular senescence by resisting senescence onset or restricting SASP to inhibit senescent cell clearance and lead to immune escape [9, 10, 11, 12]. Recent studies have revealed that long noncoding RNAs (lncRNAs) are also involved in the regulation of cellular senescence in breast cancer, with pro‐carcinogenic lncRNAs acting as key players in resisting cellular senescence [13]. LncRNA MIAT is overexpressed in breast cancer, and its inhibition triggers senescence and G1 arrest in MCF7 cells [14]. LncRNA MEG3 participates in the senescent progress of A549 and MCF7 cells induced by etoposide via the miR‐16‐5p/VGLL4 axis [15]. Silencing lncRNA SNHG6 inhibits the proliferation of SK‐BR‐3 and MDA‐MB‐231 cells by inducing apoptosis and senescence [16]. Senescence‐associated gene set consisting of lncRNAs MCF2L‐AS1, USP30‐AS1, OTUD6B‐AS1, MAPT‐AS1, PRR34‐AS1 and DLGAP1‐AS1 can assess the benefits of chemotherapy and immunotherapy for breast cancer with early diagnostic utility [17]. However, the role of lncRNAs in the senescent process of TNBC has rarely been reported, necessitating further clarification of the key regulatory lncRNAs to provide novel targets for TNBC therapy, especially for TIS strategy.

In the present study, we revealed the expression features of 279 senescence‐related genes (SRGs) in TNBC and clarified the differentially expressed lncRNAs (DElncRNAs) by bioinformatics and further identified senescence‐related lncRNAs (SRlncRNAs) by WGCNA. DElncRNAs and SRlncRNAs were interacted to obtain differentially expressed SRlncRNAs as key SRlncRNAs. A target lncRNA was locked by the interaction analysis and literature research. Cellular function and mechanistic pathway validation were performed in vitro by constructing knockdown cell lines of the target lncRNA. CIBERSORT analysis further revealed the effect of target lncRNA on immune infiltration in TNBC.

## 2 Materials and Methods

### 2.1 Data Collection and Processing

RNA sequencing (RNA‐seq) data and copy number variation (CNV) data for 1217 cases of breast invasive carcinoma (BRCA) from The Cancer Genome Atlas (TCGA) database were downloaded from the UCSC Xena database (https://xenabrowser.net/). Clinical information of TCGA‐BRCA samples was downloaded from the cBioPortal database (http://www.cbioportal.org/). The raw data type of RNA‐seq was fragments per kilobase per million (FPKM), which were used for subsequent analyses by converting to transcripts per million (TPM) expressed as log2 (TPM + 1). Numeric focal‐level CNV values were generated with ‘masked copy number segment’ files from tumour aliquots using GISTIC2.0 software. Genes with focal CNV values < −0.3 were categorised as ‘single detection’. Genes with focal CNV values > 0.3 were categorised as ‘single gain’. Genes with focal CNV values ≥ −0.3 and ≤ 0.3 were categorised as ‘normal’. TCGA‐BRCA samples were further categorised into 113 TNBC (all negative), 865 non‐TNBC (at least one positive) and 239 uncertain types based on the ER, PR and HER2 statuses provided by the clinical information. The downloaded CNV data contained 110 TNBC samples.

### 2.2 Differentially Expressed Gene Screening

CellAge database (http://genomics.senescence.info/cells) was used to download 279 SRGs identified from gene manipulation experiments [18]. Limma package in R language was used to compare the expression of 279 SRGs and all lncRNAs between TNBC (*n* = 113) and non‐TNBC (*n* = 865) groups. Differentially expressed SRGs (DESRGs) and DElncRNAs were screened based on Benjamini–Hochberg's method with thresholds of |log2 (fold change, FC)| > 1 and *p* < 0.05. Related volcano plots and heatmaps were plotted using the ggplot2 package in R language.

### 2.3 Co‐Expression Correlation Analysis

Expression correlations between DESRGs were analysed by Spearman's method based on the RNA‐seq data of 113 TNBC samples. Correlation heatmap was plotted by the pheatmap package in R language.

### 2.4 Functional and Pathway Enrichment Analysis

For Gene Ontology (GO) analysis, the subset (c5.go.bp.v7.4.symbols.gmt) from the Molecular Signatures Database (http://www. gsea‐msigdb.org/gsea/downloads.jsp) was used as the background to map the DESRGs inside of it. For Kyoto Encyclopedia of Genes and Genome (KEGG) analysis, the KEGG database (https://www.kegg.jp/kegg/rest/keggapi.html) was used to obtain the latest gene annotation, which was used as the background to map the DESRGs inside of it. GO and KEGG enrichment analyses were performed using the clusterProfiler package in R language. The minimum and maximum gene sets were set to 5 and 5000, respectively, and the terms with enriched gene counts in top 20 and *p* < 0.05 were displayed.

### 2.5 SRlncRNA Screening

SRlncRNAs were screened with the WGCNA package in R language. The steps are provided briefly as follows: (1) the lncRNAs with variance in top 50 were selected to establish the network; (2) the Person correlation of the RNA expression was calculated, and the correlation coefficient matrix was established; (3) sample clustering and exclusion of outlier samples; (4) the appropriate β soft threshold parameter (set to 3) was selected to establish the scale‐free networks by network topology analysis; (5) the correlation coefficient matrix was transformed into topological overlap matrix (TOM), and TOM was used for clustering; (6) the lncRNAs with a similar expression level were classified into the same module by the Dynamic Tree Cut algorithm; (7) the correlation between DESRGs and modules was calculated; and (8) the lncRNAs in modules with |*R*| > 0.3 and *p* < 0.05 were screened out.

### 2.6 SRlncRNA Interaction Analysis

Differentially expressed SRlncRNAs were obtained by interacting DElncRNAs with SRlncRNAs screened by WGCNA. Based on the clustering results of WGCNA, the co‐expression correlation (weight > 0.05) and expression levels of differentially expressed SRlncRNAs in the modules were visualised using Cytoscape software.

### 2.7 Gene Enrichment Analysis

A total of 113 TNBC samples were categorised into high‐ (*n* = 57) and low (*n* = 56)‐expression groups based on the median value (0.92) of the SOX9‐AS1 expression. Pathway enrichment analysis was performed for both groups using the GSEA tool (http://software.broadinstitute.org/gsea/index.jsp), and the pathways with enrichment scores (ES) in top 10 and *p* < 0.05 were displayed.

### 2.8 Immune Infiltration Analysis

Reliable assessment of immune infiltration was performed using the immunedeconv package in R language based on the sample grouping from gene enrichment analysis. The CIBERSORT algorithm was used to calculate the enrichment scores of each sample for 22 immune cells in both groups through the gglpot2 and GSVA packages in R language.

### 2.9 Cell Culture

Human breast epithelial cells (MCF10A), non‐TNBC cells (MCF7) and TNBC cells (MDA‐MB‐231, MDA‐MB‐453 and HCC1806) were provided by Guangzhou Boyao Biotechnology Co. Ltd. Cells were cultured in DMEM (Gibco, USA) containing 10% FBS (Gibco, USA) and 50 μg/mL of penicillin/streptomycin (Beyotime, China) in a humidified atmosphere at 37°C with 5% CO_2_.

### 2.10 SOX9‐AS1 Knockdown Cell Line Construction

Three short hairpin (sh) RNAs containing shSOX9‐AS1‐a, shSOX9‐AS1‐b and shSOX9‐AS1‐c were designed targeting SOX9‐AS1, and empty vector was used as the negative control (shNC). Four SOX9‐AS1 knockdown cell lines were provided by Guangzhou Boyao Biotechnology Co. Ltd. The steps are provided in brief as follows: MDA‐MB‐231 cells (5 × 10^4^ cells/mL) were seeded in 2 mL of DMEM with 10% FBS in 6‐well culture plate. After 24 h of seeding, 1 mL of transfection solution was replaced with 15 μL of SOX9‐AS1 knockdown lentiviral plasmid (1.38 × 10^8^ U/mL, MOI = 10), 200 μL of Poly⁃Brene co‐transfectant (50 μg/mL) and 785 μL of high‐sugar DMEM complete culture medium. After 8 h of replacing the transfection solution, the high‐sugar DMEM complete culture medium containing 2 μg/mL of puromycin was replaced again. After 48 h of culturing, the lentiviral transfection efficiency was observed under a fluorescence microscope. Sequences of three shRNAs are summarised in Table S1.

### 2.11 Plasmid Construction and Transfection

The pIRES2‐ZsGreen1‐SOX9‐AS1 plasmid was synthesised by Guangzhou General Biomedical Technology Co. Ltd. (Guangzhou, China) for overexpressing SOX9‐AS1 with an empty plasmid as NC. Cells were seeded into 6‐well plate at 1 × 10^6^ cells/mL with 2 mL of serum medium. Lipofectamine 2000 (Thermo Fisher, USA) was used for transfection according to the manufacturer's instructions.

### 2.12 Cell Proliferation Assay

Cells (1 × 10^3^ cells/well) were seeded in a 96‐well plate and cultured by adding 100 μL of DMEM complete culture medium. At 6, 12, 24 and 48 h, 10 μL of CCK‐8 reagent (Beyotime, China) was added and the cells were continued incubating for 2 h. The OD values of cells at 450 nm were detected by a microplate reader, and the cell proliferation curves were plotted.

### 2.13 Cell Cycle Assay

Cells were collected after 48 h of culturing, washed twice with pre‐chilled PBS, and resuspended by adding a pre‐chilled 75% ethanol and fixed at −20°C overnight. Cells were collected by centrifugation, and cell suspension (1 × 10^6^ cells/tube) was prepared. Then, 500 μL of PBS solution with 50 μg/mL of PI, 100 μg/mL of RNaseA and 0.2% Triton X‐100 reagents (Beyotime, China) were added and incubated at 4°C protected from light for 30 min. Finally, the cell fluorescence was detected at 488 nm, and the cycle ratios were analysed by flow cytometry with NovoExpress software.

### 2.14 Cell Apoptosis Assay

Cells were collected after 48 h of culturing, and the cell suspension (1 × 10^6^ cells/tube) was prepared with binding buffer. Then, 5 μL of Annexin V‐APC and 10 μL of 7‐AAD reagents (Beyotime, China) were added, vortexed, and incubated at room temperature (25°C) protected from light for 15 min. Cell suspension was prepared by resuspending 485 μL of a pre‐chilled binding buffer in each tube. Finally, the cell fluorescence was detected at 488 nm, and the apoptosis rates were analysed by flow cytometry with NovoExpress software.

### 2.15 Cell Migration Assay

Cells (5 × 10^5^ cells/well) were seeded in 6‐well plate and cultured by adding 2 mL of DMEM complete culture medium. Parallel lines were drawn at the bottom of the culture plate with a pipette tip, and the fallen cells were rinsed with PBS. The migrating cells at 0 and 48 h were imaged, and the migration rates were calculated by Image J software.

### 2.16 Cell Invasion Assay

A dual‐chamber system with an 8‐μm pore was used in this assay, and the upper chamber was pre‐added with 80 μL of matrix solution and incubated at 37°C for 1 h. Cells (5 × 10^4^ cells/well) were seeded in the upper chamber of the inserts with 200 μL of serum‐free DMED culture medium, and the lower chamber was filled with 500 μL of DMEM complete culture medium. After 48 h of invading, the inserts were fixed with 100% methanol and then stained with 0.1% crystal violet (Beyotime, China). The invading cells on the inserts were imaged and counted by Image J software.

### 2.17 Cell Viability Assay

Cells (1 × 10^3^ cells/well) were seeded in a 96‐well plate with 100 μL of serum medium for 24 h. Then, 5, 10, 20 and 40 μM of tamoxifen (TAM) (MCE, USA) were added and cultured for 24 h. The OD values of cells at 450 nm were detected with 10 μL of CCK‐8 reagent by a microplate reader.

### 2.18 Senescence‐Associated β‐Galactosidase (SA‐β‐Gal) Staining

Cellular senescence was detected using SA‐β‐gal staining kit (Beyotime, China). Cells were washed twice with PBS and fixed in 4% formaldehyde (Beyotime, China) at room temperature for 15 min. After adding 1 mL of freshly prepared SA‐β‐gal staining solution (1 mg/mL) containing 10 μL of solution A, 10 μL of solution B, 930 μL of staining solution C and 50 μL of X‐Gal solution for overnight incubation, cells were observed and imaged under an optical microscope.

### 2.19 Quantitative Reverse Transcriptase PCR (qRT‐PCR) Assay

Cells were washed with PBS thrice, and total RNA was extracted with Trizol reagent (BioTeke, China). After determining the concentration and purity of RNA samples, RNA reverse transcription was performed according to the steps of reverse transcription kit (Vazyme, China). The synthetic cDNAs were used as a template for fluorescence detection using SYBR Green qPCR detection kit (Biosharp, China). The reaction procedure is given in brief as follows: pre‐denaturation at 95°C for 30 s, followed by 40 cycles of denaturation at 95°C for 5 s, annealing at 60°C for 30 s, and extension at 72°C for 1 min. Relative quantification was performed by the 2^−∆∆*CT*^ method, and β‐actin was used as the internal reference. Primer sequences of six genes (SOX9‐AS1, IL‐1α, IL‐1β, IL‐6, IL‐8 and β‐actin) are summarised in Table S2.

### 2.20 Western Blot (WB) Assay

Cells were washed with PBS thrice and lysed in RIPA lysis buffer (Beyotime, China) supplemented with 0.1% protease‐inhibitor (Beyotime, China). Forty micrograms of isolated proteins were separated on 12% SDS‐PAGE gels and electrotransferred to PVDF membranes. Then, they were incubated for 14 h with primary antibodies against GSK‐3β (1:1000, Abcam, USA), β‐catenin (1:1000, Abcam, USA) and GAPDH (1:1500, Abcam, USA). The PVDF membrane was washed with a TBST buffer (Beyotime, China) and incubated in an HRP‐conjugated secondary antibody for 2 h. Protein bands were detected using a chemiluminescent luminol enhancer solution (YEASEN, China) after the TBST wash. The gel imaging system was used for imaging analysis, and the grey‐scale values of protein bands were calculated using Image J software.

### 2.21 Statistical Analysis

All experiments were performed in triplicate. Statistical analysis was performed by SPSS software. Experimental data were presented as mean ± SD. Comparison between multiple groups was made using one‐way ANOVA (Tukey's post hoc test). *p* < 0.05 was considered statistically significant.

## 3 Results

### 3.1 Aberrant Expression of SRGs in TNBC

**FIGURE 1:**
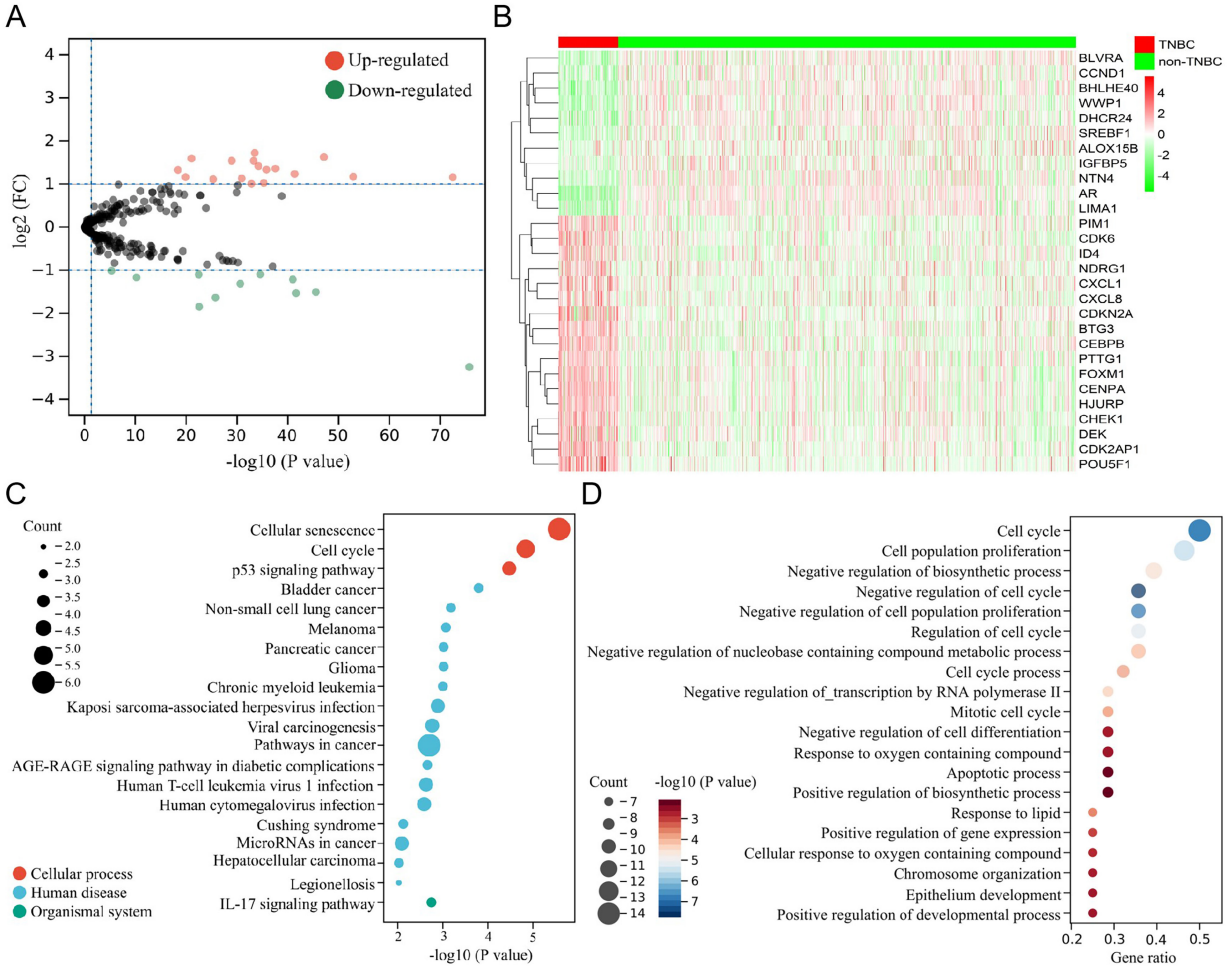
Expression and functional features of DESRGs in TNBC. (A) Volcano plot displaying the expression of 28 DESRGs between TNBC (*n* = 113) and non‐TNBC (*n* = 865) samples. (B) Heatmap displaying the expression of 28 DESRGs in each TNBC and non‐TNBC sample. (C) KEGG analysis for the enrichment of 28 DESRGs in the cellular process, human disease and organismal system. (D) GO analysis for the enrichment of 28 DESRGs in the biological process.

To understand the role of 279 SRGs in TNBC, TCGA‐BRCA samples were categorised into TNBC and non‐TNBC groups based on ER, PR and HER2 statuses, and their expression was further compared. Twenty‐eight SRGs were differentially expressed in TNBC, with 11 down‐regulated (AR, BHLHE40, WWP1, LIMA1, BLVRA, DHCR24, CCND1, NTN4, SREBF1, IGFBP5 and ALOX15B) and 17 up‐regulated (POU5F1, CDK2AP1, CENPA, CEBPB, BTG3, HJURP, CHEK1, CDK6, CXCL1, FOXM1, DEK, PIM1, CDKN2A, PTTG1, ID4, NDRG1 and CXCL8) (Figure 1A,B). CNV analysis revealed that nine DESRGs (BHLHE40, BTG3, CCND1, CDK2AP1, CDKN2A, CHEK1, DEK, DHCR24 and PIM1) significantly increased in expression at a single gain onset and decreased at a single detection onset (Figure S1A–I). Co‐expression correlation analysis revealed mostly positive correlation among DESRGs, with FOXM1 correlating the strongest to CENPA and CHEK1 (*R* = 0.77) (Figure S2). These DESRGs were mainly enriched in cell fate processes like senescence, cycle, proliferation and apoptosis and were also involved in the regulation of various cancers like bladder cancer, non‐small cell lung cancer, melanoma and pancreatic cancer, as well as in the regulation of tumour‐associated pathways like p53 and IL‐17 signals (Figure 1C,D). These results suggest that SRGs play an important regulatory role in TNBC progression, especially in cancer cell fate.

### 3.2 SOX9‐AS1 Is a Key SRlncRNA in TNBC

**FIGURE 2:**
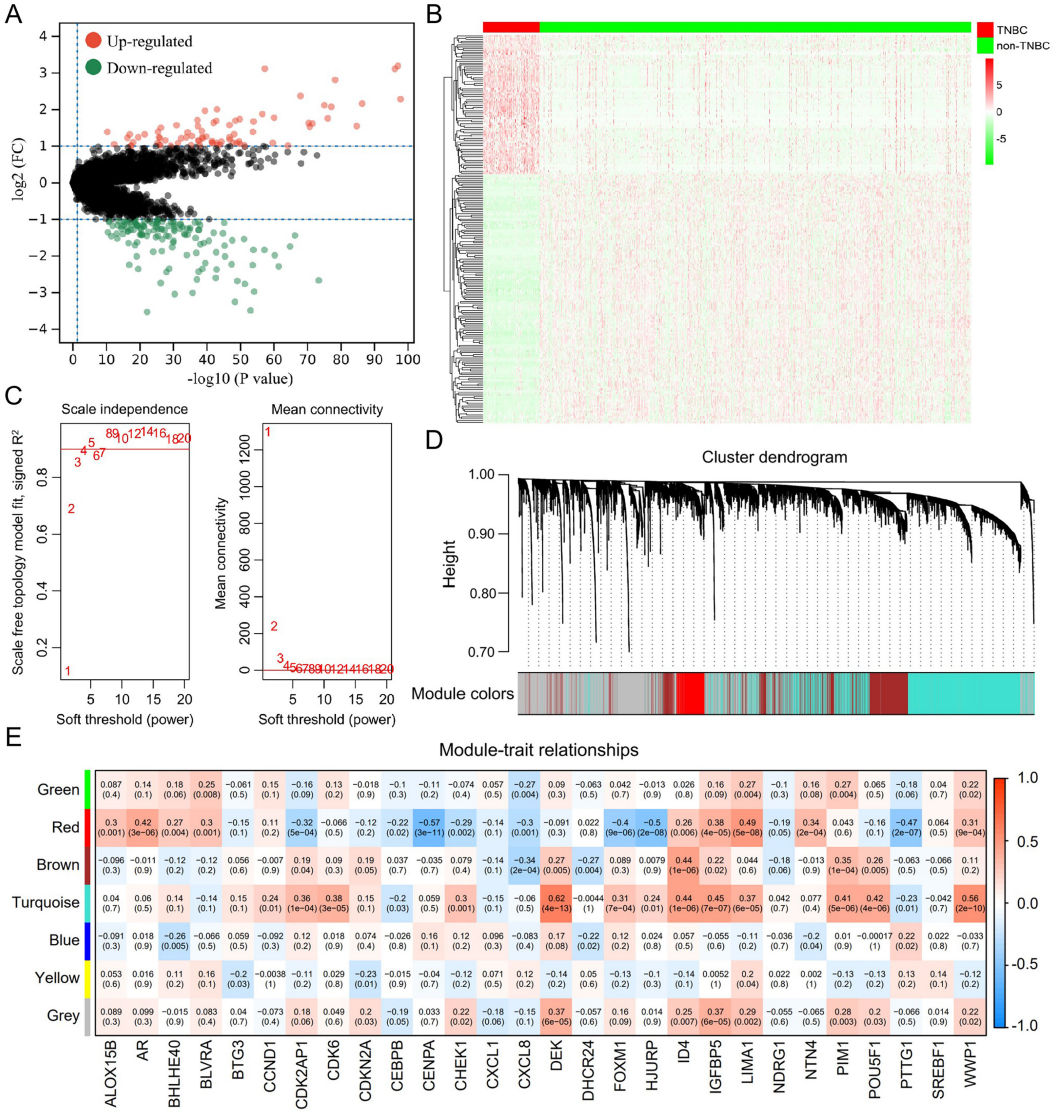
DElncRNA screening and SRlncRNA identification in TNBC. (A) Volcano plot displaying the expression of 212 DElncRNAs between TNBC (*n* = 113) and non‐TNBC (*n* = 865) samples. (B) Heatmap displaying the expression of 212 DElncRNAs in each TNBC and non‐TNBC sample. (C) Scale independence and mean connectivity in TNBC samples. (D) Cluster dendrogram and modules before merging in TNBC samples. (E) Pearson correlation analysis of merged modules for SRlncRNAs in TNBC samples.

**FIGURE 3:**
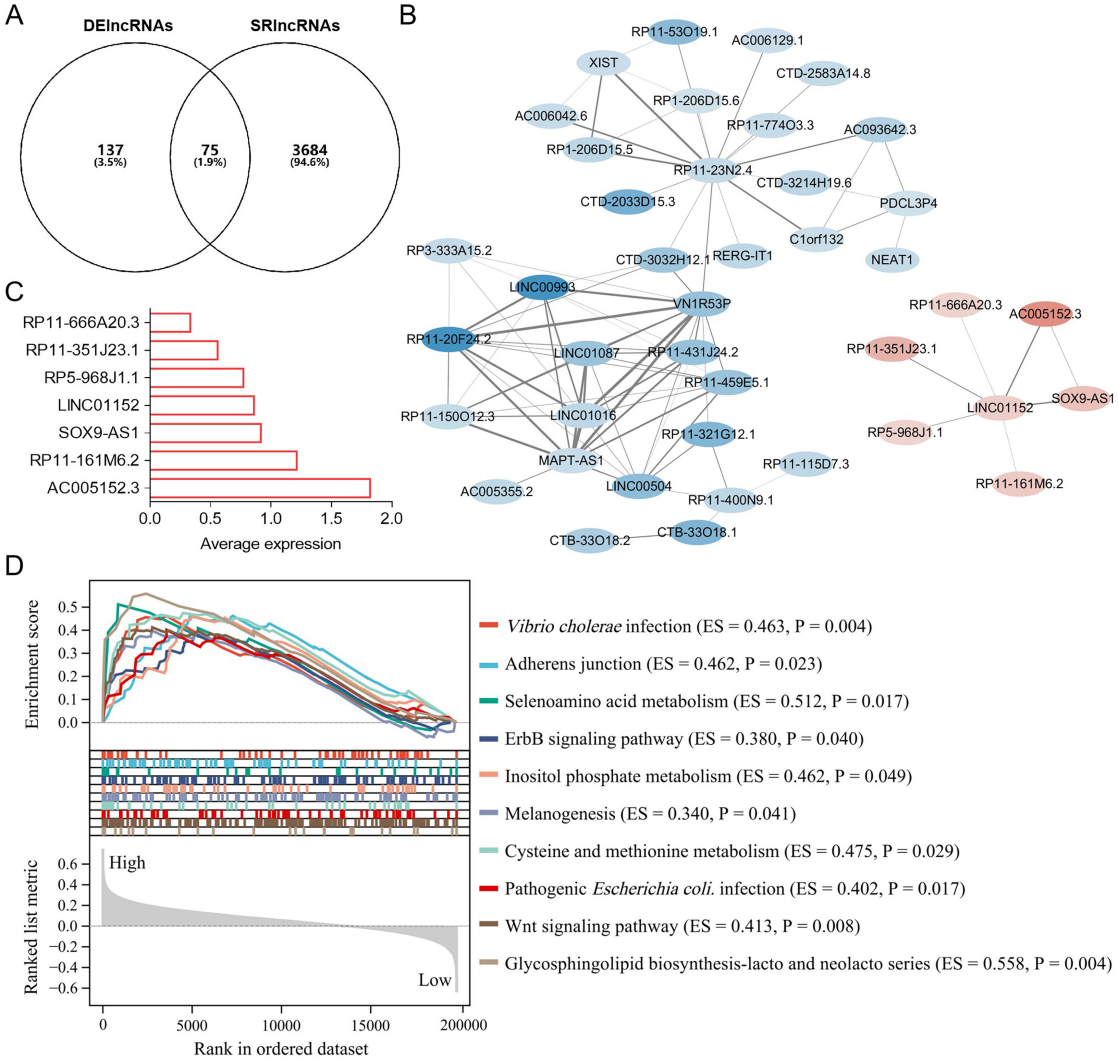
Identification of a key SRlncRNA (SOX9‐AS1) in TNBC. (A) Venn diagram displaying the interaction of 212 DElncRNAs with 3759 SRlncRNAs. (B) Interaction network of 75 differentially expressed SRlncRNAs and visualisation of the fraction with weight > 0.05 (red lncRNAs indicate up‐regulation in TNBC and blue lncRNAs indicate down‐regulation in TNBC). (C) Average expression of seven SRlncRNAs (SOX9‐AS1, LINC01152, AC005152.3, RP11‐161M6.2, RP5‐968J1.1, RP11‐351J23.1 and RP11‐666A20.3) in TNBC samples. (D) Pathway enrichment of SOX9‐AS1 in high‐ (*n* = 57) and low (*n* = 56)‐expression samples of TNBC.

To obtain SRlncRNAs, 212 DElncRNAs with 136 down‐regulated and 76 up‐regulated were first screened in TNBC and non‐TNBC groups, which were highly associated with TNBC progression (Figure 2A,B). The correlation between DESRGs and all lncRNAs was further established by WGCNA, in which lncRNAs with similar expression levels were included in the same module, and the grey module indicated the lncRNAs that were not assigned. In other six modules (green, red, brown, turquoise, blue and yellow), 3759 lncRNAs with |*R*| > 0.3 and *p* < 0.05 were screened out, which were highly associated with senescence and characterised as SRlncRNAs (Figure 2C–E). Finally, 75 differentially expressed SRlncRNAs were obtained by interacting SRlncRNAs with DElncRNAs, which mediated tumour senescence regulation and were involved in TNBC progression (Figure 3A). SRlncRNA interaction analysis revealed that the red gene cluster containing SOX9‐AS1, LINC01152, AC005152.3, RP11‐161M6.2, RP5‐968J1.1, RP11‐351J23.1 and RP11‐666A20.3 existed independently and were highly expressed in TNBC (Figure 3B,C). Literature research on these seven SRlncRNAs confirmed that SOX9‐AS1 exerted pro‐carcinogenic effects in various tumours, including TNBC. However, the involvement of SOX9‐AS1 in regulating senescence and its mediating mechanistic pathway in TNBC has not been reported, which aroused our interest. Gene enrichment analysis further revealed that multiple biological mechanisms were highly enriched in TNBC patients with high SOX9‐AS1 expression, like adherens junction, selenoamino acid metabolism and ErbB and Wnt signalling pathways (Figure 3D). These results suggest that SOX9‐AS1 closely correlates with senescence regulation in TNBC and targeting SOX9‐AS1 may exert an effective anti‐TNBC effect.

### 3.3 SOX9‐AS1 Knockdown Exerts Anti‐TNBC Effects In Vitro

**FIGURE 4:**
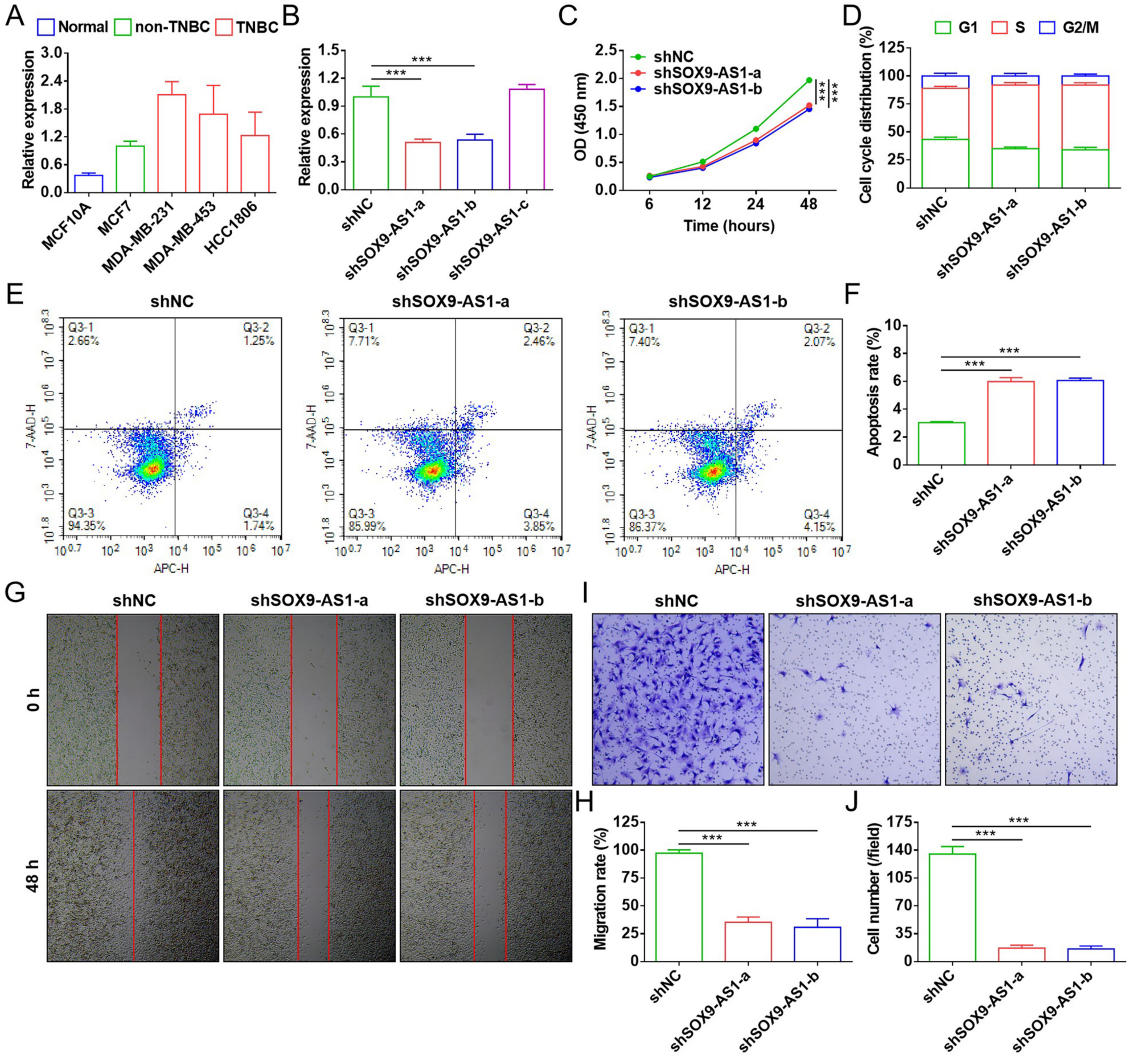
Effect of SOX9‐AS1 knockdown on the MDA‐MB‐231 cell function. (A) Expression of SOX9‐AS1 transcript in MCF10A, MCF7, MDA‐MB‐231, MDA‐MB‐453 and HCC1806 cells at 48 h. (B) Expression of SOX9‐AS1 transcript in shNC, shSOX9‐AS1‐a, shSOX9‐AS1‐b and shSOX9‐AS1‐c cells at 48 h. (C) OD values of cells at 6, 12, 24 and 48 h. (D) Statistics of cell cycle distribution at 48 h. (E) Cell apoptosis levels at 48 h. (F) Statistics of cell apoptosis levels. (G) Cell migration levels at 48 h. (H) Statistics of cell migration levels. (I) Cell invasion levels at 48 h. (J) Statistics of cell invasion levels (***p < 0.001).

To prove the pro‐carcinogenicity of SOX9‐AS1 on TNBC, cellular function validation was performed in vitro. The expression level of SOX9‐AS1 in different breast cancer cells was detected by qRT‐PCR. Compared with normal and non‐TNBC cells, the SOX9‐AS1 expression was higher in TNBC cells, with the highest expression in MDA‐MB‐231 cells (Figure 4A). SOX9‐AS1 was further knocked down in MDA‐MB‐231 cells by lentiviral transduction, and two knockdown cell lines (shSOX9‐AS1‐a and shSOX9‐AS1‐b) were successfully identified by qRT‐PCR for comparison (Figure 4B). Functional experiments revealed that SOX9‐AS1 knockdown significantly inhibited cell proliferation, blocked cell cycle and promoted cell apoptosis (Figure 4C–F). In addition, SOX9‐AS1 knockdown suppressed the cell malignant phenotypes, including significant inhibition of cell migration and invasion (Figure 4G–J). SOX9‐AS1 overexpression promoted SOX9‐AS1 knockdown MDA‐MB‐231 cell proliferation, migration and invasion and inhibited apoptosis (Figure S3A–G). These results indicate that SOX9‐AS1 plays an important mediating role in TNBC progression as an oncogene.

### 3.4 SOX9‐AS1 Knockdown Facilitates Cellular Senescence in TNBC via Inhibiting Wnt Signalling Pathway

**FIGURE 5:**
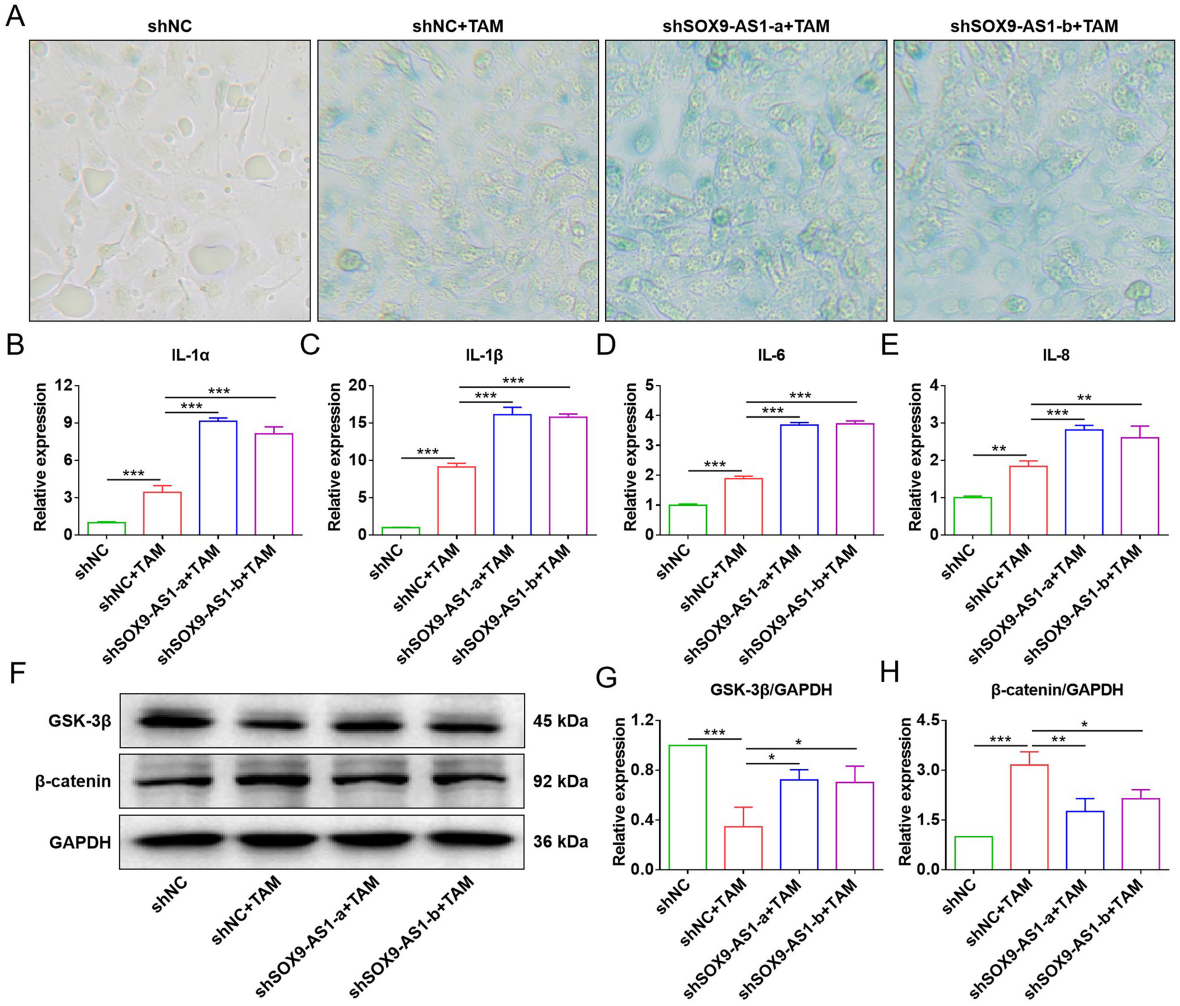
Effect of SOX9‐AS1 knockdown on TAM‐induced senescence and Wnt signalling pathway in MDA‐MB‐231 cells. (A) SA‐β‐gal staining in cells at 24 h. (B) Expression of IL‐1α transcript in cells at 24 h. (C) Expression of IL‐1β transcript in cells at 24 h. (D) Expression of IL‐6 transcript in cells at 24 h. (E) Expression of IL‐8 transcript in cells at 24 h. (F) Expression levels of GSK‐3β, β‐catenin and GAPDH proteins in cells at 24 h. (G) Statistics of GSK‐3β/GAPDH protein levels. (H) Statistics of β‐catenin/GAPDH protein levels (*p < 0.05; **p < 0.01; ***p < 0.001).

Furthermore, the relationship between SOX9‐AS1 and senescence was explored by inducing a senescence model in vitro. Given that TAM is commonly used for the clinical therapy of recurrent or metastatic breast cancer, it was chosen to induce senescence in MDA‐MB‐231 cells. Pre‐experiments showed that 5 μM of TAM treatment for 24 h inhibited cell viability and withdrew TAM for 24 h, which could induce cellular senescence (Figure S4A–C). Therefore, pre‐treatment with 5 μM of TAM for 24 h was used to establish the cellular senescence model. SA‐β‐gal staining revealed that TAM successfully induced senescence in shNC cells, while shSOX9‐AS1‐a and shSOX9‐AS1‐b cells showed more severe degrees (Figure 5A). Consistent with this result, SOX9‐AS1 knockdown significantly facilitated the transcriptional expression of SASP factors IL‐1α, IL‐1β, IL‐6 and IL‐8 (Figure 5B–E), since Wnt signals exerted an important regulatory role in mediating cellular senescence and was highly enriched in TNBC patients with high SOX9‐AS1 expression. Thus, we speculated that SOX9‐AS1 could mediate TNBC senescence via regulating Wnt signalling pathway. The activation status of pathway was measured by detecting two key transducer molecules of Wnt signals, GSK‐3β and β‐catenin. WB results demonstrated that TAM induced the activation of the Wnt signalling pathway (GSK‐3β down‐regulation and β‐catenin up‐regulation), while SOX9‐AS1 knockdown attenuated the induction (Figure 5F–H). These findings suggest that SOX9‐AS1 involves in the senescence process of TNBC via regulating the Wnt signalling pathway.

### 3.5 Low SOX9‐AS1 Expression Enhances Immune Infiltration in TNBC

**FIGURE 6:**
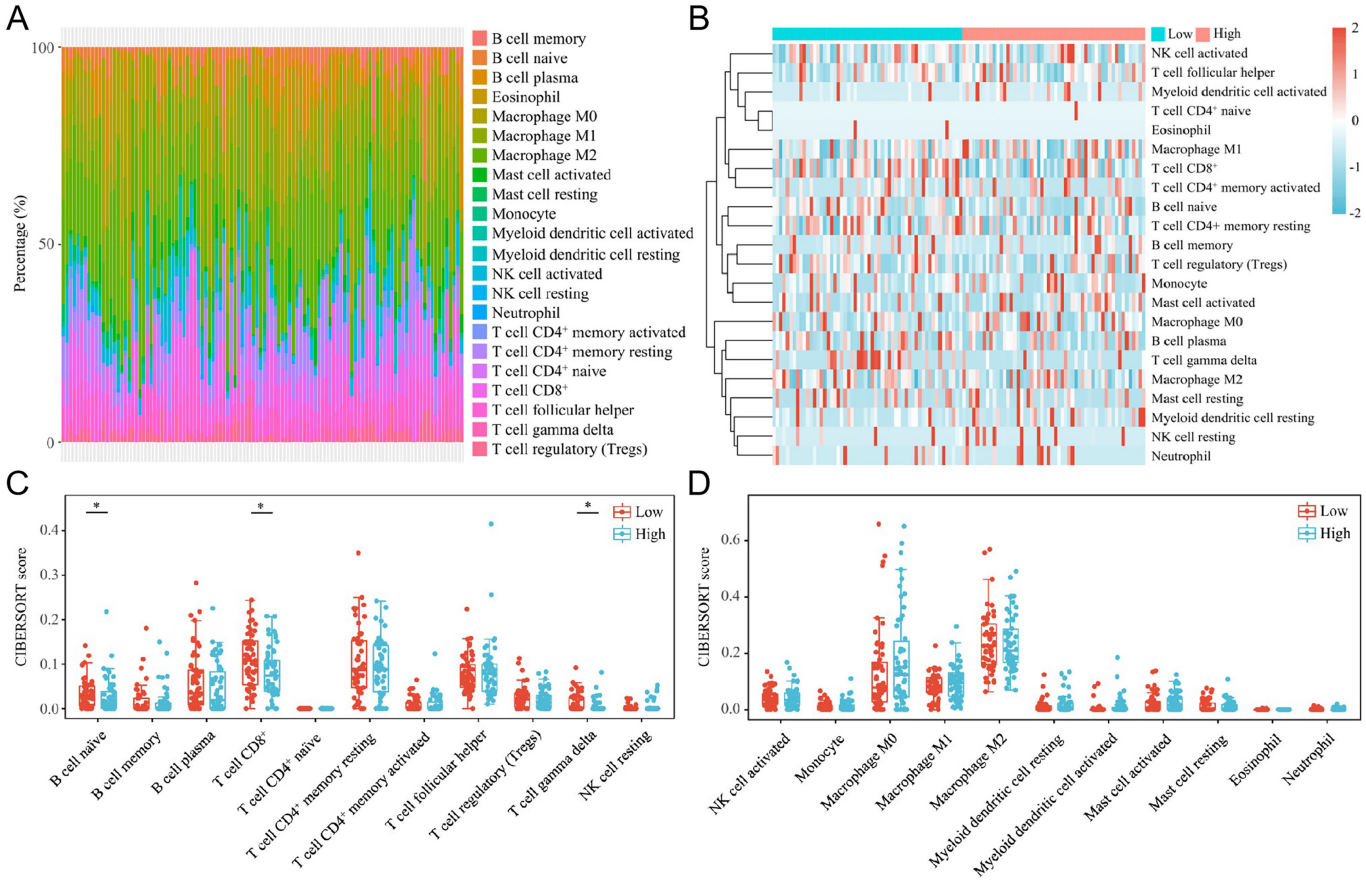
Effect of SOX9‐AS1 expression on immune infiltration in TNBC. (A) Distribution of 22 immune cells in each TNBC sample (*n* = 113). (B) Heatmap displaying the expression of 22 immune cells in high‐ (*n* = 57) and low (*n* = 56)‐SOX9‐AS1 expression samples of TNBC. (C) and (D) Expression statistics of 22 immune cells in high‐ and low‐SOX9‐AS1 expression samples of TNBC (*p < 0.05).

Cellular senescence, a biological process in which cells stop dividing and lose their proliferative capacity, exerts a tumour‐suppressive effect to some extent. Moreover, SASP factors secreted by senescent cells can recruit immune cells to enhance immune clearance. However, whether the pro‐carcinogenic SOX9‐AS1 affects the tumour immune microenvironment (TIME) alterations of TNBC is still unclear, necessitating further revelation of its immunoregulatory role. Immune infiltration analysis revealed that TIME of TNBC was infiltrated with various immune cells, with macrophages, T cells and B cells accounting for a major portion of the total (Figure 6A). TNBC samples were further categorised into low‐ and high‐expression groups based on the median value of SOX9‐AS1 expression, and the infiltration levels of 22 different immune cells were displayed in heatmap (Figure 6B). The immunosuppressive effect of SOX9‐AS1 was revealed by scoring the infiltration of 22 different immune cells. Low SOX9‐AS1 expression accompanied a high infiltration of tumour‐killing immune cells, including naïve B cells, CD8^+^ T cells and γδ T cells (Figure 6C,D), and, conversely, high SOX9‐AS1 expression suppressed immune infiltration. These results suggest the feasibility of a therapeutic strategy for inducing cellular senescence and enhancing immune clearance in TNBC via SOX9‐AS1 inhibition.

## 4 Discussion

Clinical consequences of TNBC patients vary widely, which is associated with their high heterogeneity [19]. Studies have found that senescence plays an important mediating role in TNBC heterogeneity, and facilitating cellular senescence can restrict tumour heterogeneity to some extent, which is an effective anti‐TNBC strategy [20]. However, the regulation of TNBC senescence is currently unclear, which requires further revelation of their relationship. Thus, we started with 279 identified SRGs to observe their expression features in TNBC for establishing the association between senescence and TNBC progression.

Twenty‐eight SRGs were revealed to be aberrantly expressed in TNBC by bioinformatics, of which 11 were down‐regulated (AR, BHLHE40, WWP1, LIMA1, BLVRA, DHCR24, CCND1, NTN4, SREBF1, IGFBP5 and ALOX15B) and 17 were up‐regulated (POU5F1, CDK2AP1, CENPA, CEBPB, BTG3, HJURP, CHEK1, CDK6, CXCL1, FOXM1, DEK, PIM1, CDKN2A, PTTG1, ID4, NDRG1 and CXCL8). Partial DESRGs have been confirmed to regulate TNBC progression. CircWAC/miR‐142/WWP1 form a competing endogenous RNA network to regulate the PI3K/AKT signalling activity in TNBC cells [21]. LncRNA HOST2 knockdown inhibits the proliferation of TNBC cells via regulating let‐7b/CDK6 axis [22]. USP7 inhibition induces p53‐independent tumour growth suppression in TNBC via destabilising FOXM1 [23]. CXCL8 facilitates the survival and paclitaxel resistance of TNBC [24]. The aberrant expression of these DESRGs is closely correlated with CNV, and their interactive association may collectively contribute to TNBC progression [25, 26, 27]. Functional enrichment revealed the primary involvement of these DESRGs in the regulation of cancer cell fate, further demonstrating the close association between senescence and TNBC progression.

Due to the complex heterogeneity of TNBC, finding novel TIS targets remains a challenge. In recent years, senescence regulation has been confirmed to mediate TNBC heterogeneity, in which lncRNAs exert key functional roles with promising biomarkers [28]. Thus, we further resolved the critical lncRNAs involved in the regulation of TNBC senescence. Firstly, 212 DElncRNAs highly correlated with TNBC were screened. Further correlation of DESRGs with all lncRNAs was established by WGCNA, yielding 3759 SRlncRNAs. Then, 75 differentially expressed SRlncRNAs were obtained by the interaction of SRlncRNAs with DElncRNAs. Finally, interaction analysis identified an independent gene set containing SOX9‐AS1, LINC01152, AC005152.3, RP11‐161 M6.2, RP5‐968 J1.1, RP11‐351 J23.1 and RP11‐666A20.3, which were highly expressed in TNBC. SOX9‐AS1, LINC01152 and RP11‐351 J23.1 have been reported to be involved in cancer progression, with SOX9‐AS1 acting as a pro‐carcinogen in various tumours more widely [29, 30, 31]. In intrahepatic cholangiocarcinoma, high SOX9‐AS1 expression is related to shorter overall survival and recurrence‐free survival and promotes tumour growth and metastasis [32]. In hepatocellular carcinoma, the SOX9‐AS1/miR‐5590‐3p/SOX9 positive feedback loop drives tumour growth and metastasis through the Wnt pathway [33]. However, the role of SOX9‐AS1 in TNBC has rarely been investigated, especially in mediating senescence regulation remains in gap. Subsequent gene enrichment analysis revealed pathway enrichment for high SOX9‐AS1 expression in TNBC and identified the overactivity of the Wnt signalling pathway, a key regulatory signal for cellular senescence in various cancers [34]. These results suggest that SOX9‐AS1 may mediate TNBC senescence via regulating the Wnt signalling pathway.

To verify our speculation, related cellular function and mechanistic pathway validation were performed in vitro. Compared with normal and non‐TNBC cells, SOX9‐AS1 was more highly expressed in TNBC cells, especially in MDA‐MB‐231 cells, suggesting its pro‐carcinogenic role. Moreover, SOX9‐AS1 knockdown significantly inhibited cell growth and malignant phenotype, while overexpression had the opposite effect. Cellular senescence was induced by acting with low‐dose TAM, which revealed that SOX9‐AS1 knockdown accelerated cellular senescence and facilitated the transcriptional expression of SASP factors IL‐1α, IL‐1β, IL‐6 and IL‐8. Physiologically, senescence often accompanies pro‐inflammatory SASP [35]. These inflammatory factors can cause a feed‐forward signalling cascade that induces healthy cells to enter senescence, while Wnt signals interrupt this cascade via inhibiting SASP factors [36]. Consistent with this, TAM‐induced cellular senescence caused the activation of the Wnt signalling pathway, while SOX9‐AS1 knockdown attenuated this induction, emphasising the importance of Wnt signals against TNBC senescence. These findings demonstrate that SOX9‐AS1 knockdown inhibits the adaptive resistance to Wnt signals induced by TAM‐induced cellular senescence, which consequently facilitates TNBC senescence and death. Therefore, SOX9‐AS1 is a promising anti‐TNBC target, especially for providing novel insights into TIS strategy.

SASP has a bidirectional regulation, which mainly relies on the microenvironment of senescent cells. In positive regulation, IL‐1, IL‐6 and IL‐8 in SASP facilitate the growth arrest of senescent cells through autocrine action and accelerate senescence by acting together with p53 and pRb in the tumour‐suppressor pathways [37]. Additionally, certain inflammatory factors and chemokines in SASP recognise and remove senescent cells by recruiting immune cells [38]. These are the main therapeutic mechanisms of the TIS strategy. In negative regulation, senescent cells can destroy the normal tissue structure and promote epithelial–mesenchymal transition, such as senescent mesenchymal fibroblasts secrete SASP factors to degrade fibrous tissues and MMP secreted by senescent cells can disrupt the function of mammary epithelial cells [39, 40]. Thus, how to promote SASP toward a positive regulation is the critical part of the TIS strategy. Our previous studies demonstrated that SOX9‐AS1 knockdown facilitated senescence and inflammatory SASP in MDA‐MB‐231 cells, and cellular function also showed significant anti‐tumour effects. Further immune infiltration analysis revealed that low SOX9‐AS1 expression accompanied a high infiltration of tumour‐killing immune cells, including naïve B cells, CD8^+^ T cells and γδ T cells. Mechanistically, SOX9‐AS1 appears to modulate immune cell infiltration by regulating the expression of immunomodulatory molecules within the tumour microenvironment. High levels of SOX9‐AS1 may up‐regulate VEGF‐A expression, promoting angiogenesis and contributing to an immunosuppressive milieu that hinders the infiltration of cytotoxic immune cells. Additionally, SOX9‐AS1 might influence the secretion of chemokines such as CCL2 and CXCL12, which recruit immunosuppressive cells like regulatory T cells (Tregs) and myeloid‐derived suppressor cells (MDSCs), further inhibiting anti‐tumour immune responses. By altering these signalling pathways, SOX9‐AS1 reduces the infiltration and activity of CD8+ T cells and γδ T cells, which are crucial for tumour cell killing. Furthermore, SOX9‐AS1 may down‐regulate the expression of adhesion molecules on endothelial cells, impeding the transmigration of immune cells into the tumour tissue [41]. These findings suggest that SOX9‐AS1 plays a pivotal role in shaping the immune landscape of TNBC by creating a suppressive environment that facilitates tumour progression. Therefore, targeting SOX9‐AS1 could reverse immunosuppression, enhance the infiltration of effector immune cells, and potentiate anti‐tumour immunity in TNBC. TIS renders cancer cells highly immunogenic, making them very efficient in triggering protective CD8‐dependent anti‐tumour immune responses. SASP can enhance anti‐tumour immune responses via activating IFN signal transduction, augmenting MHC class I molecules and stimulating senescence‐specific auto‐peptides of CD8^+^ T cells, which efficiently activate dendritic cells and antigen‐specific CD8^+^ T cells [41]. And γδ T, as the immune cell with the strongest tumour‐killing ability, not only exerts a scavenging effect via releasing cytotoxic substances like perforin, granzyme and cytokines, but also synergistically activates other immune cells like B cells, αβ T cells and NK cells, which collectively achieve the anti‐tumour effect [42]. In addition, humoral immunity is closely related to senescence regulation. Senescence process accompanies the reduction of naïve B cells and the expansion of memory B cells that exhibit senescence‐associated phenotype, and the mobilisation of the B cell‐mediated immune regulatory system is an effective way to remove senescent cells [43]. These studies support the feasibility for the targeted inhibition of SOX9‐AS1 to exert immune clearance by enhancing immune infiltration in TNBC, providing a novel direction for adjuvant therapy of TIS.

In conclusion, SOX9‐AS1 acts as an oncogene that resists TNBC senescence via regulating the Wnt signalling pathway and possesses a potent immunoregulatory function.

## Author Contributions

**Xuan Ye:** conceptualization (equal), investigation (equal), methodology (equal), writing – original draft (equal). **Yi Cen:** conceptualization (equal), investigation (equal), methodology (equal), writing – original draft (equal). **Quan Li:** conceptualization (equal), investigation (equal), methodology (equal), writing – original draft (equal). **Yuan‐Ping Zhang:** investigation (equal). **Qian Li:** investigation (equal). **Jie Li:** conceptualization (equal), funding acquisition (equal), project administration (equal), supervision (equal).

## Conflicts of Interest

The authors declare no conflicts of interest.

## Supporting information


**Figure S1.** Co‐expression correlation among 28 DESRGs in TNBC.

**Figure S2.** CNV of nine DESRGs in TNBC. (A) CNV of BHLHE40, (B) CNV of BTG3, (C) CNV of CCND1, (D) CNV of CDK2AP1, (E) CNV of CDKN2A, (F) CNV of CHEK1, (G) CNV of DEK, (H) CNV of DHCR24 and (I) CNV of PIM1.

**Figure S3.** Effect of SOX9‐AS1 overexpression on the SOX9‐AS1 knockdown MDA‐MB‐231 cell function. (A) OD values of cells at 6, 12, 24 and 48 h. (B) Cell apoptosis levels at 48 h. (C) Statistics of cell apoptosis levels. (D) Cell migration levels at 48 h. (E) Statistics of cell migration levels. (F) Cell invasion levels at 48 h. (G) Statistics of cell invasion levels (*p < 0.05; **p < 0.01; ***p < 0.001).

**Figure S4.** Identification for the optimal senescence‐inducing dose of TAM in MDA‐MB‐231 cells. (A) Effect of 5, 10, 20 and 40 μM TAM on cell viability at 24 h. (B) OD values of cells at 24 h. (C) SA‐β‐gal staining after pre‐treatment with 5 μM TAM for 24 h and after withdrawal of TAM for 24 h.

**Table S1.** Sequences of three shRNAs targeting SOX9‐AS1.

**Table S2.** Primer sequences of six genes.

## Data Availability

The data presented in this study are available on request from the corresponding authors.
