# Supplementary figures and images for: Immunosuppressive SOX9‐AS1 Resists Triple‐Negative Breast Cancer Senescence Via Regulating Wnt Signalling Pathway
Source: J Cell Mol Med. 2024 Nov 17;28(22):e70208. doi: 10.1111/jcmm.70208 (PMC11569622; doi:10.1111/jcmm.70208)

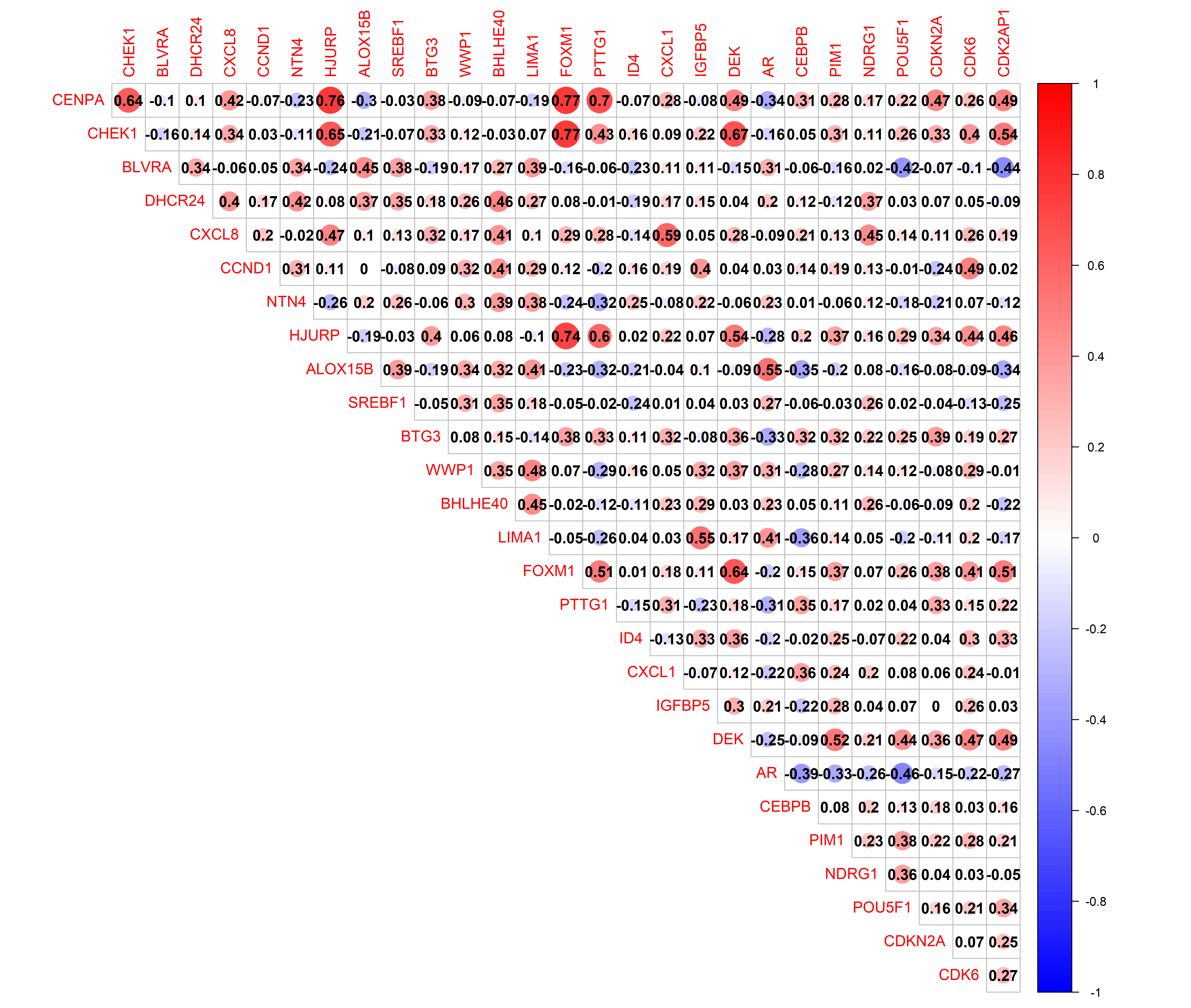

Supplement: Supplementary file 1 — Figure S1. Co‐expression correlation among 28 DESRGs in TNBC. [file JCMM-28-e70208-s001.png]

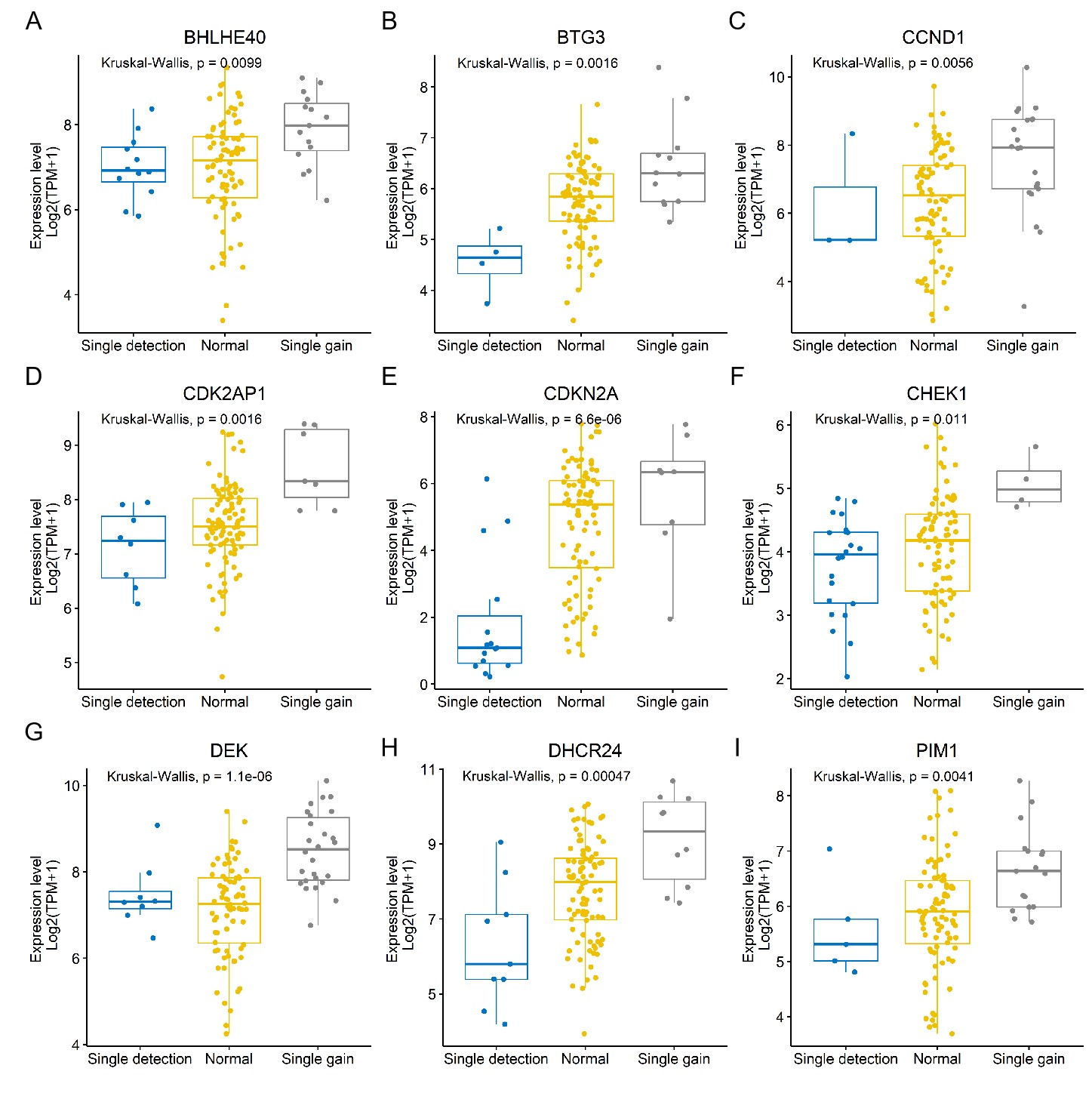

Supplement: Supplementary file 2 — Figure S2. CNV of nine DESRGs in TNBC. (A) CNV of BHLHE40, (B) CNV of BTG3, (C) CNV of CCND1, (D) CNV of CDK2AP1, (E) CNV of CDKN2A, (F) CNV of CHEK1, (G) CNV of DEK, (H) CNV of DHCR24 and (I) CNV of PIM1. [file JCMM-28-e70208-s006.jpg]

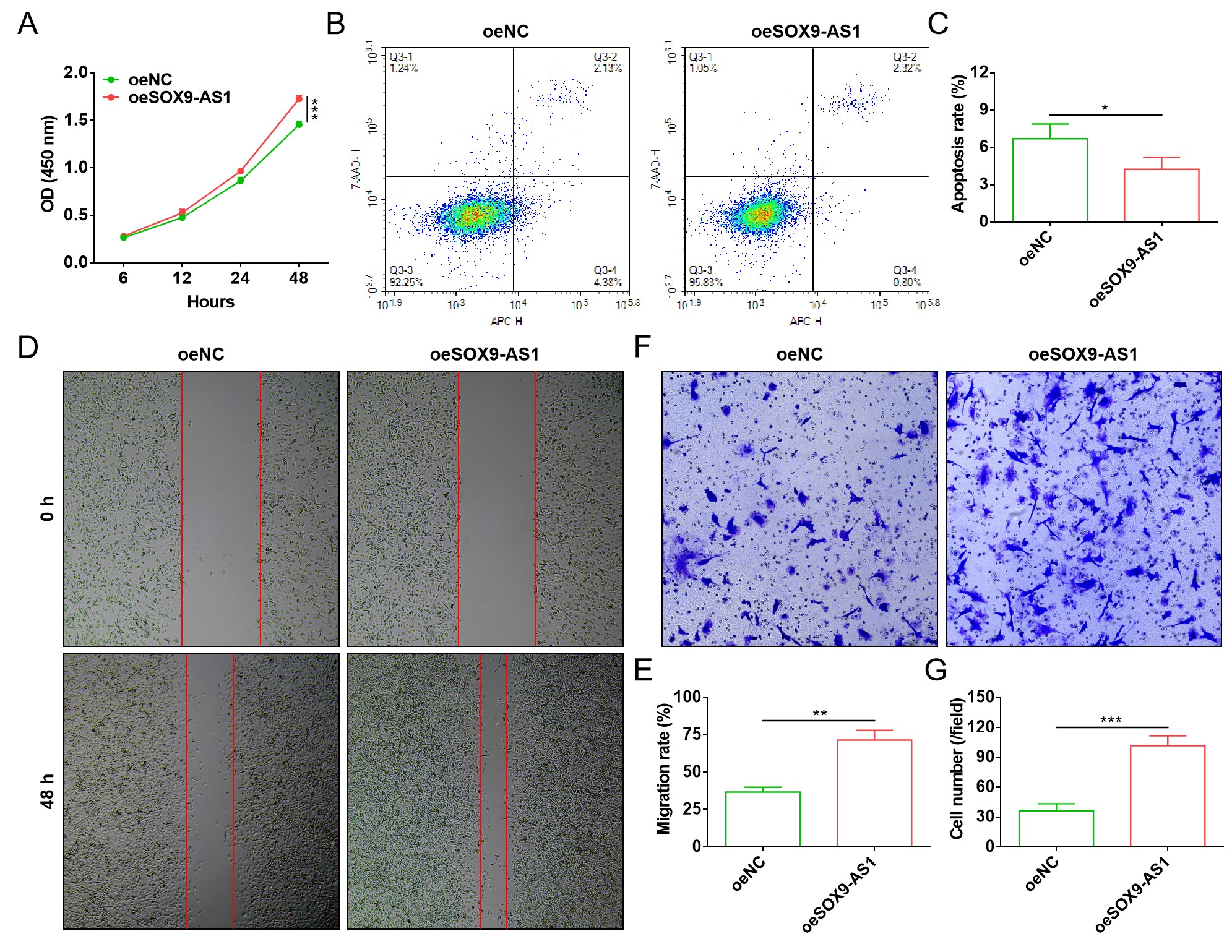

Supplement: Supplementary file 3 — Figure S3. Effect of SOX9‐AS1 overexpression on the SOX9‐AS1 knockdown MDA‐MB‐231 cell function. (A) OD values of cells at 6, 12, 24 and 48 h. (B) Cell apoptosis levels at 48 h. (C) Statistics of cell apoptosis levels. (D) Cell migration levels at 48 h. (E) Statistics of cell migration levels. (F) Cell invasion levels at 48 h. (G) Statistics of cell invasion levels (*p < 0.05; **p < 0.01; ***p < 0.001). [file JCMM-28-e70208-s002.jpg]

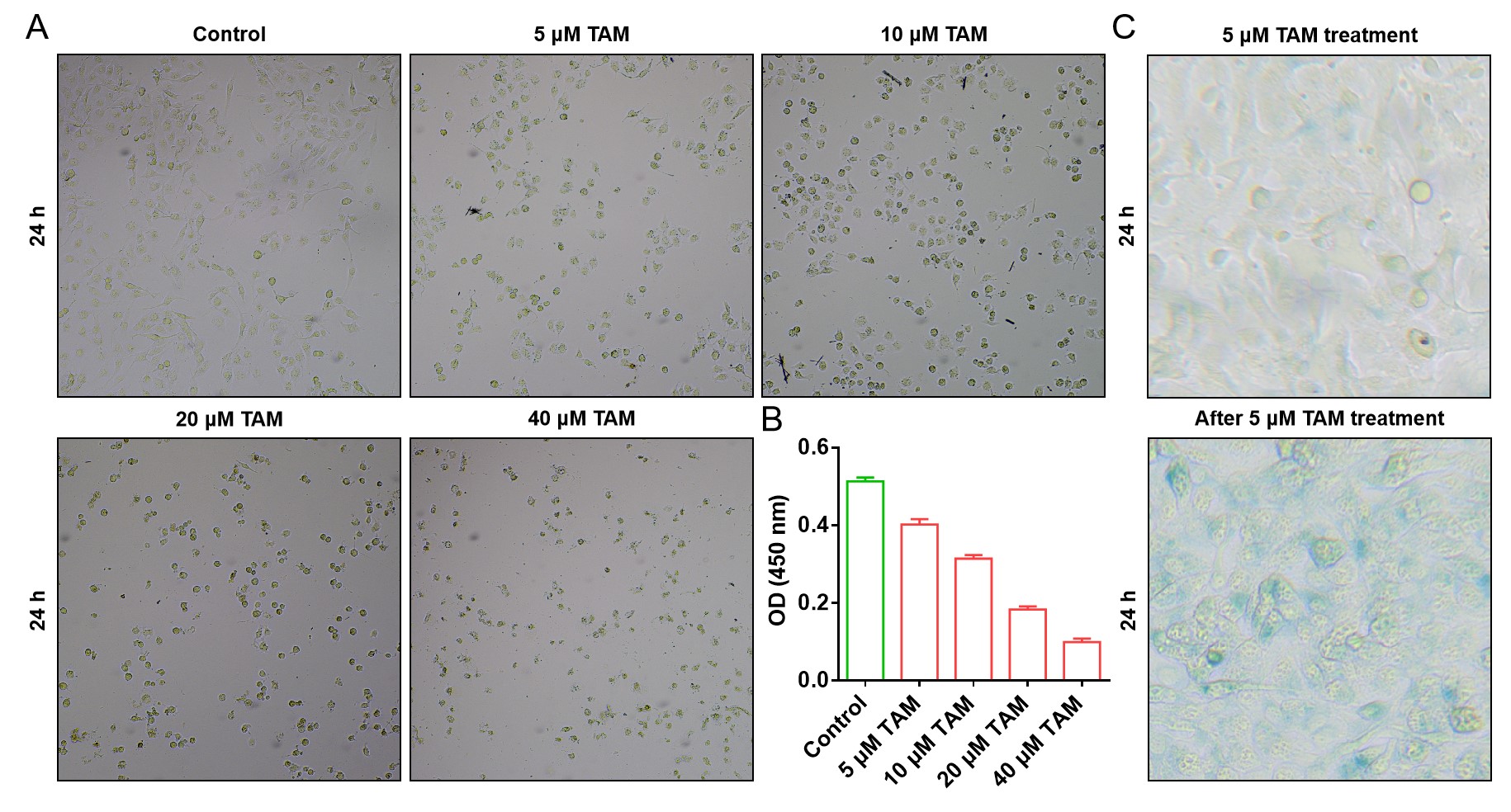

Supplement: Supplementary file 4 — Figure S4. Identification for the optimal senescence‐inducing dose of TAM in MDA‐MB‐231 cells. (A) Effect of 5, 10, 20 and 40 μM TAM on cell viability at 24 h. (B) OD values of cells at 24 h. (C) SA‐β‐gal staining after pre‐treatment with 5 μM TAM for 24 h and after withdrawal of TAM for 24 h. [file JCMM-28-e70208-s004.jpg]
