# Supplementary material for: Immunosuppressive SOX9‐AS1 Resists Triple‐Negative Breast Cancer Senescence Via Regulating Wnt Signalling Pathway
Source: J Cell Mol Med. 2024 Nov 17;28(22):e70208. doi: 10.1111/jcmm.70208 (PMC11569622; doi:10.1111/jcmm.70208)
Supplement: Supplementary file 5 — Table S1. Sequences of three shRNAs targeting SOX9‐AS1. [file JCMM-28-e70208-s003.docx]

Table S1. Sequences of 3 shRNAs targeting SOX9-AS1

| **shRNAs** | **5'-Stem** | **Loop** | **Stem-3'** |
| --- | --- | --- | --- |
| shSOX9-AS1-a (sense) | CAAAGGAGCTAAAGAAGAA | CTCGAG | TTCTTCTTTAGCTCCTTTG |
| shSOX9-AS1-a (anti-sense) | CAAAGGAGCTAAAGAAGAA | CTCGAG | TTCTTCTTTAGCTCCTTTG |
| shSOX9-AS1-b (sense) | GGACATTCCTCAAGATCAA | CTCGAG | TTGATCTTGAGGAATGTCC |
| shSOX9-AS1-b (anti-sense) | GGACATTCCTCAAGATCAA | CTCGAG | TTGATCTTGAGGAATGTCC |
| shSOX9-AS1-c (sense) | GGTGGTGGTCATTGTGCTA | CTCGAG | TAGCACAATGACCACCACC |
| shSOX9-AS1-c (anti-sense) | GGTGGTGGTCATTGTGCTA | CTCGAG | TAGCACAATGACCACCACC |
