# Supplementary material for: Immunosuppressive SOX9‐AS1 Resists Triple‐Negative Breast Cancer Senescence Via Regulating Wnt Signalling Pathway
Source: J Cell Mol Med. 2024 Nov 17;28(22):e70208. doi: 10.1111/jcmm.70208 (PMC11569622; doi:10.1111/jcmm.70208)
Supplement: Supplementary file 6 — Table S2. Primer sequences of six genes. [file JCMM-28-e70208-s005.docx]

Table S2. Primer sequences of 6 genes

| **Genes** | **Primer sequences (5' to 3')** |
| --- | --- |
| SOX9-AS1 (forward) | TGTTCACCTTGGATGAGAGCAG |
| SOX9-AS1 (reverse) | GACCCCAAGCAAAGTTCCCAA |
| IL-1α (forward) | TCATCCTGAATGACGCCCTC |
| IL-1α (reverse) | CCCATGTCAAATTTCACTGCTT |
| IL-1β (forward) | AGGATATGGAGCAACAAGTGGT |
| IL-1β (reverse) | TTTCAACACGCAGGACAGGT |
| IL-6 (forward) | GGTCCAGTTGCCTTCTCCCT |
| IL-6 (reverse) | CCAGTGCCTCTTTGCTGCTT |
| IL-8 (forward) | GCTCTGTGTGAAGGTGCAGTT |
| IL-8 (reverse) | TTCTGTGTTGGCGCAGTGTG |
| β-actin (forward) | TGGCACCCAGCACAATGAA |
| β-actin (reverse) | CTAAGTCATAGTCCGCCTAGAAGCA |
